# Heat Flow Estimation in Polymer Films during Orientational Drawing at the Local Heater

**DOI:** 10.3390/polym16162267

**Published:** 2024-08-10

**Authors:** Liubov Myasnikova, Yuri Kurakin, Vladimir Hilarov, Vyacheslav Marikhin, Maria Narykova, Elena Ivan’kova

**Affiliations:** 1 Laboratory of Physics of Strength, Ioffe Institute, Politekhnicheskaya 26, 194021 St. Petersburg, Russia; vladimir.hilarov@mail.ioffe.ru (V.H.); v.marikhin@mail.ioffe.ru (V.M.); maria.narykova@mail.ioffe.ru (M.N.); 2 Computational Physics Laboratory, Ioffe Institute, Politekhnicheskaya 26, 194021 St. Petersburg, Russia; yurii.kurakin@mail.ioffe.ru; 3 Institute of Macromolecular Compounds, V.O., Bolshoy pr. 31, 199004 St. Petersburg, Russia; ivelen@mail.ru

**Keywords:** multi-stage zone drawing, numerical simulation of heat flows, thermal fluctuation theory of strength, neck formation, defect generation, scanning and optical microscopy

## Abstract

The optimization of the process of polymer film orientational drawing using the local heater was investigated. One of the problems with this technology is that the strength of the resulting fibers differs significantly from the theoretical estimates. It is assumed that one of the reasons is related to the peculiarity of this technology, when at the point of drawing the film is heated only on one side, which creates a temperature difference between the sides of the film in contact with the heater and the non-contact sides of the film in the air. Estimates show that even a small temperature difference of just 1 °C between these surfaces leads to a significant difference in the rate of plastic deformation of the corresponding near-surface layers. As a consequence, during hardening, in the stretching region, tensile stress is concentrated on the “cold” side of the film, and this effect can presumably lead to the generation of more defects overthere. It has been suggested that defects arising during first stage of hardening, namely, neck formation, can serve as a trigger for the formation of defects such as kink bands on the “cold” side with further orientational strengthening due to plastic deformation of the resulting fibrillar structure, at the boundaries of which microcracks are formed, leading to rupture of the oriented sample. The numerical calculation of heat propagation due to heat conduction in the film from the local surface of the heater is carried out and the temperature distribution along the thickness and width of the film during drawing is found. The temperature difference in the heated layer of the film between the contact and non-contact sides with the heater was calculated depending on the thickness of the film and the speed of its movement along the heater. It was found that the most homogeneous temperature distribution over the film thickness, which is required, by default, for the synchronous transformation of the unoriented initial folded lamellar structure into a fibrillar structure, is observed only for films with a thickness of less than 50 μm. The calculation allows us to scientifically justify the choice of orientation drawing speed and optimal thickness of the oriented polymer film, which is extremely important, for example, for obtaining super-strong and high-modulus UHMWPE filaments used in products for various purposes: from body armor to sports equipment and bioimplants,

## 1. Introduction

Partially crystalline polymers have a complicated multi-level hierarchical morphology, highly influencing the mechanical properties of polymeric materials. When the polymer is crystallized from undisturbed melts or solutions, its strength is small and several orders of magnitude lower than the theoretical estimates. This is explained by the complexity of the morphological units when only a part of the molecules carries the load applied to the sample. To increase the strength of the polymeric material, it is necessary to increase the number of chains bearing the load. A typical method for the production of ultra-strong high-modulus polymer fibers and films is orientation drawing (deformation hardening), which consists of a uniaxial drawing of non-oriented films or fibers by the applied load at temperatures below the polymer melting temperature. As it was found, orientation drawing represents the competition of two processes: strengthening due to the molecular chain alignment in the drawing direction and loss of strength which occurs in accordance with Zhurkov’s theory due to thermal fluctuation scissions of the molecules under the load which is less than their tensile strength. S.N. Zhurkov was the first [1,2,3,4,5] to propose the idea that the sample rupture under mechanical stress is not a catastrophic event with immediate body breakdown into two parts, but the result of the accumulation of macromolecule scissions affected by thermal fluctuations. It is not the load applied to the loaded body, but the thermal fluctuations that help the atom overcome the potential well deformed by the load Molecular breaks are accompanied by the formation of macroradicals, which leads to the formation of micro- and macrocracks. Their merging ultimately leads to the propagation of the main crack and failure of the oriented sample. [1,2,3,4,5]. The kinetics of the process is described by the Arrhenius relation:

(1) $$\tau =\tau_{0}\exp\left(\frac{U_{0}-\gamma_{\tau }\sigma }{kT}\right)$$

where *τ* is the sample lifetime under the load, *τ*_0_ is the period of atomic oscillations around the equilibrium position (~10^−13^ s, *U*_0_ is the chemical bond energy, *γ_τ_* is the structurally sensitive factor, *σ* is the applied load, *k* is the Boltzmann constant, and *T* is the temperature [1].

According to Equation (1), the lifetime of the sample under mechanical load and high temperature decreases exponentially.

**Figure 1:**
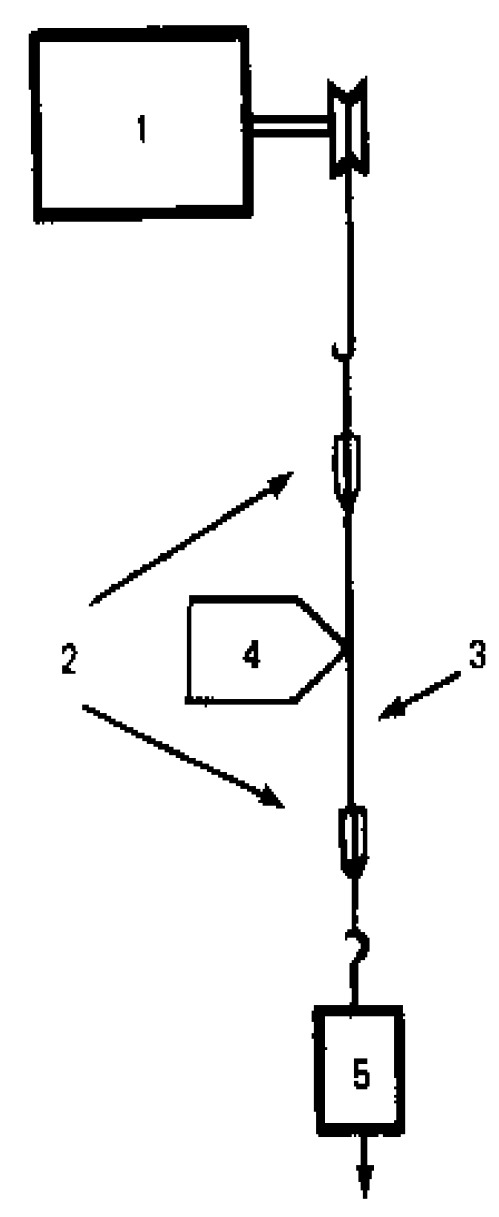
A scheme of a high-temperature multi-stage zone drawing: The oriented film 3, fixed in clamps 2, is fed to the local heater using motor 1 and moves along heater 4 under the action of applied load 5, stretching and strengthening.

With the aim of diminishing the negative effects, Ioffe Institute developed a multi-stage zone orientation drawing method that allows for minimizing the oriented sample residence time in “dangerous” conditions (at high temperatures and under high mechanical load). This method is shown schematically in Figure 1.

The movement of the film along the heater is repeated several times with increasing temperature and load [5,6,7,8,9].

The size of the contact zone is selected so that at a given feed rate and load, the time the specimen remains in hazardous conditions is minimized. The orientation drawing should be carried out in a stepwise manner for the following reasons: Firstly, because during drawing, two processes that are fundamentally different in their physical nature occur. Each of them must be carried out at the optimal temperature for this process.

**Figure 2:**
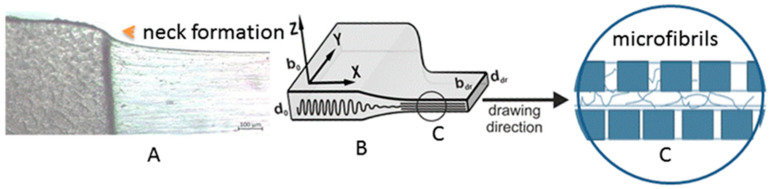
An optical micrograph of the neck region formed during the drawing process (**A**) and a schematic of the reorganization of the original lamellar structure into a fibrillar structure in the neck (**B**). A schematic representation of the microfibril structure: alternation of crystalline and disordered regions along the fibrils (**C**).

A schematic model of the processes taking place is presented in Figure 2.

The first stage of orientation drawing is the neck formation (Figure 2A), accompanied by narrowing (b_o_→b_dr_) and thinning (d_o_→d_dr_) of the oriented film (Figure 2B). As has been shown in numerous research papers, any initial morphology of non-oriented polymer films or fibers subjected to orientational drawing is transformed into the fibrillar one during necking (Figure 2C) [7,9,10,11,12,13,14,15,16,17,18,19,20,21,22,23,24,25]. To minimize the number of molecular chain scissions, the neck formation must be carried out at a temperature when the van der Waals interactions between the molecules in the crystallites are significantly reduced due to thermal expansion [6,7,8,22]. The value of these interactions depends on the length of the molecular fold, which, in turn, is determined by the temperature of polymer crystallization from solution or melt [13,24].

The next stage of drawing is plastic deformation, which occurs due to the sliding of newly formed fibrils relative to each other. The plastic deformation of the fibrillar structure is performed at temperatures notably higher than the necking temperature.

Secondly, because the structural sensitive γ-factor decreases with an increasing draw ratio, the data were obtained, in particular, by analyzing the shifts of the weak wing of the doublet 720–730 cm^−1^ depending on the temperature and stress applied to the PE sample [26]. Then, immediately applying a large load to a poorly oriented sample with a large γ-factor value reduces the lifetime of the sample in accordance with Zhurkov’s formula (1), and, therefore, the load should be increased gradually. It is worth noting that a stepwise increase in load at the same heater temperature may lead to mechanical glass transition which prevents further plastic deformation. To continue drawing, it is necessary to increase the temperature of the heater. In the last drawing stages, the orientation temperature might be even higher than the melting point of the polymer in the non-loaded state.

Thus, taking into account the above statements and the fact that orientation hardening is a combination of two processes, 1. orientation of molecular chains along the drawing direction and 2. molecular scissions by the Zhurkov thermal fluctuation oscillation mechanism, it is possible to develop an optimal temperature and force scenario to maximize the draw ratio and, consequently, the high mechanical properties of the oriented polymer.

It should be noted, however, that not only the correct choice of temperature–force multi-stage zone drawing scenario ensures a high degree of draw ratio but also an important role in achieving the maximum degree of draw ratio is played by the initial morphology of the non-oriented material and the nature of its transition to the fibrillar structure. At the same time, there is still no consensus on the understanding of this transition. Two different models proposed for the mechanism of the structure rearrangement have been discussed for a long time.

A. Peterlin [14,15] proposed a rheological model and suggested that when a tensile force is applied, the lamellas of fold chain crystals disintegrate into nanoblocks, from which thin anisodiametric microfibrillar formations are then built. In this case, microfibrils will have, along their lengths, periodic structures of alternate folded blocks interconnected with a very small number of tie molecules. According to Peterlin’s approach, the size of the intrafibrillar crystalline regions will be determined by the size of the molecular folds in the original lamellae.

Another model was proposed by Kobayashi who suggested the recrystallization mechanism of structural rearrangement in the neck region [13,27]. According to this model, the tensile deformation leads to the complete unfolding of molecular folds in the original lamellae, which is followed by the crystallization of unfolded oriented segments of macromolecules into new fibrils. Since segments of macromolecules contain various conformational defects, they cannot crystallize along the entire length of the macromolecule. This leads to the creation of a longitudinal long-period structure in microfibrils, consisting of crystalline and disordered regions, as in the Peterlin model. The fundamental difference between these models is the number of tie molecules in the intrafibrillar disordered regions. In Kobayashi’s model, almost all (or nearly all) molecular segments are tie molecules. They go from one crystalline region through disordered regions to the neighboring crystalline region, and the crystallites do not contain folds. In this model, the value of the long period does not depend on the thickness of the original lamellas. It is controlled by the thermodynamics of crystallization at the drawing temperature.

We generally share Kobayashi’s approach to structural recrystallization [28]. At the same time, we believe that from a physical point of view, it would be more correct to describe the radical restructuring of the initial morphology into a fibrillar structure during neck formation in terms of the solid-phase transition from folded-chain crystals (FCCs) to extended-chain crystals (ECCs), via (as we suppose) the intermediate liquid crystalline state; keep in mind, however, that the unfolding of molecular folds does not occur instantly. It has been shown by many authors that the initial stages of deformation of crystalline stems (prior to unfolding of the lamella) are responsible for such modes of deformation such as twinning, phase transitions of the martensitic type, sliding of the lamellas along each other, changes in stem tilting with respect to lamellae surfaces, etc. [7,8,22,23,29,30,31]. These deformation modes are controlled by the orientation drawing temperature and parameters of the initial morphology (crystallinity, regularity of molecular folds, lamella thickness, number and conformation of tie molecules connecting lamellas, etc. [7,10,11,12,13,14,15,16,17,18,19,20,21,22,23,32,33]) and occur in a thin transition nanolayer between the original morphology and newly formed fibrillar.

**Figure 3:**
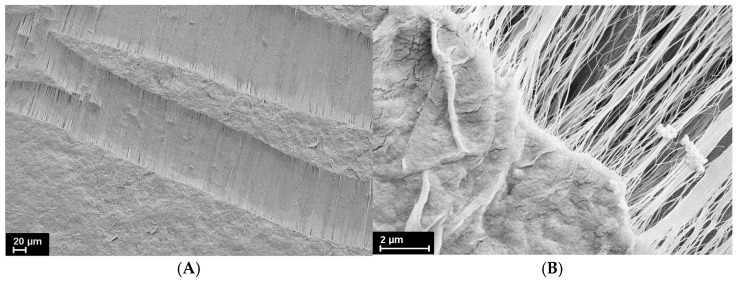
An SEM micrograph of the neck formation in the gel-crystallized UHMWPE at low (**A**) (×500) and higher (**B**) (×2000)] magnifications.

As an example, Figure 3 presents a sharp transition from folded lamellas to oriented fibrils during neck formation upon stretching of polyethylene xerogel.

This transition occurs in other semicrystalline polymers as well [7,9,11,12,20].

As noted above, the intrafibrillar disordered regions are formed by tie molecules passing along a fibril from one crystallite to the neighboring one. In poorly oriented polymers at low draw ratios, the majority of tie molecules have no flat trans-zigzag conformation. They have various conformational defects, such as kinks, double kinks, jogs, and other irregular conformations, which determine the poor mechanical properties of the oriented polymer. The taut tie molecules only bear the external load and determine the strength of the oriented material, which is higher with the greater number of load-bearing chains [7,34]. During stretching, Newtonian forces act in the transverse direction, which leads to the occurrence of shear stresses inside the fibrils as they slip. The shear forces cause conformational defects to migrate along the fibrils through the intrafibrillar crystalline regions to the ends of the molecules where they annihilate. This increases the number of load-bearing chains and, consequently, the mechanical characteristics of the oriented material.

The current state of the structural mechanics of semicrystalline polymers and the evolution of ideas about the mechanism of plasticity of solid polymers after the 1950s are described in detail in [34].

Despite the large number of experimental studies and theoretical works concerned with the study of polymer plasticity, there is still much room for further progress in understanding this phenomenon. The purpose of this work was not to add another stone to the fundamental knowledge of plasticity. As described above, many different physical processes occur during the reorganization of the original isotropic folded lamellae into a uniaxially oriented fibrillar structure. All of them depend on the external orienting load and temperature according to the exponential law. In this connection, it becomes relevant to evaluate the uniformity of temperature distribution in the oriented film. The main purpose of this work was to evaluate the temperature distribution over the thickness of a polymer film subjected to orientation drawing using a local heater and to estimate the temperature difference between the contacting and non-contacting sides of the film as a function of its thickness since the inhomogeneity of the temperature distribution over the film thickness can affect the mechanical properties of the resulting oriented polymer. These evaluations have not been previously performed for orientation drawing with the use of a local heater and are of great importance for manufacturers, as they are the basis for the scientifically justified selection of oriented drawing parameters. To visualize the possible morphological consequences of the temperature difference between the contact and non-contact sides of the oriented films in the neck region, microscopic studies of a melt-crystallized polyethylene film were performed as an example. The temperature distribution in the film as it passes through the local heater will be evaluated by numerical simulation of heat transfer in said film of a constant thickness and that of the variable thickness after the formation of the neck.

## 2. Materials and Methods

### 2.1. Objects of Study

For the study, reactor powder of a linear HDPE with a molecular weight of 160,000 g/mole, synthesized on a metallocene catalyst (rac-(Me)2Si (Ind)2ZrCl2/methylalumoxane) at a temperature of 70 °C at the N.N. Semenov Federal Research Center for Chemical Physics of the Russian Academy of Sciences, was chosen.

Preparation of the Samples

Non-oriented HDPE films were produced by compression molding at a pressure of 60 MPa, at a temperature of 150 °C in a Carver press (USA) for 15 min, followed by cooling to 125 °C under pressure and rapid cooling to room temperature after releasing pressure. Then, the films were cut into 1 mm wide strips and subjected to multi-stage zone drawing on a local heater.

### 2.2. Methods

#### 2.2.1. Microscopy

The neck formation in the films subjected to the orientation drawing was studied using a Zeiss AXIO vert.A1 optical microscope equipped with a digital camera Axiocam 208.

The oriented HDPE films were studied using both a stereoscope optical microscope SOPTOP SZN71 as well as a Zeiss Supra 55VP scanning electron microscope. To avoid charge accumulation when scanning samples with an electron probe, the particles were placed on a conductive substrate, and a thin layer of platinum was deposited onto them in a Q150T ES (Quorum Technologies Ltd. Judges HousLewes RoadLaughton East SusseBN8 6BN. UK)

#### 2.2.2. Numerical Simulation Method

If thermal expansion of the film material during heating is neglected, the motion of the film can be considered as the flow of an incompressible liquid in a channel of varying cross-sections. The computational domain is fixed relative to the heater. The X-axis is aligned along the film, and the Z-axis is perpendicular to the heater.

For a film with a constant cross-section, the channel resembles a rectangle, and the material velocity remains uniform at all points.

In simulating the heating of the film after the neck formation, the geometry of the calculation region follows the shape of the neck observed in experiments.

Additionally, several assumptions were accepted. The shape of the neck was considered symmetric with respect to the Y and Z axes. The stretching of the material occurs uniformly across the film’s cross-section, and the distribution of material velocity in the absence of vortex flows can be calculated based on the neck geometry and the law of mass conservation. The influence of crystallinity and the anisotropy of the thermal conductivity coefficient were also neglected.

Heat fluxes are described by the heat conduction equation completed by the term of convective heat transfer due to film moving.

(2) $$\frac{\partial T}{\partial t}+\nabla uT=\frac{\lambda }{\rho \cdot c}\nabla^{2}T$$

Here, *λ* = 0.418 W/m/K is the polymer heat conductivity, *ρ* = 980 kg/m^3^ is the polymer density, *c* = 1760 J/kg/K is the polymer heat capacity, and *u* [m/s] is the film moving speed.

In solving the equation, an original solver developed in our institute was used. It is based on the finite volume method [35,36,37,38], one of the most widely used methods in continuum dynamics, utilized in globally renowned hydrodynamic software packages such as FLUENT, CFX, STAR–CD, FINE (NUMECA), CFD–ACE, etc.

**Figure 4:**
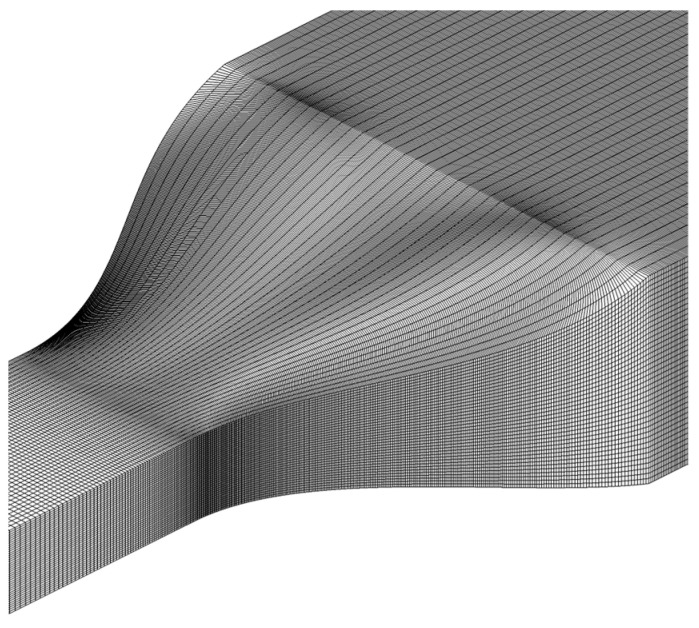
An example of a computational grid that follows the shape of a neck.

The simulation was carried out on a structured 3D computational grid (Figure 4).

Boundary conditions are the following when the film comes into contact with a local heater, the surface temperature is equal to the heater temperature; at the ends of the film there are conditions of continuity of heat flow; on the film surface external to the atmosphere, heat fluxes are calculated using tabulated empirical heat transfer constants between a solid surface and air [39].

## 3. Results

**Figure 5:**
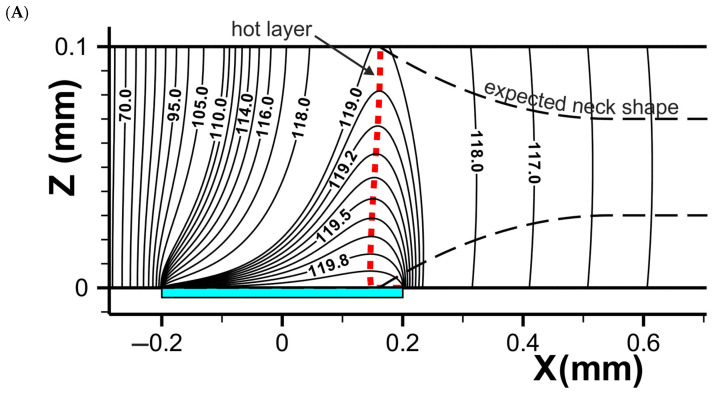

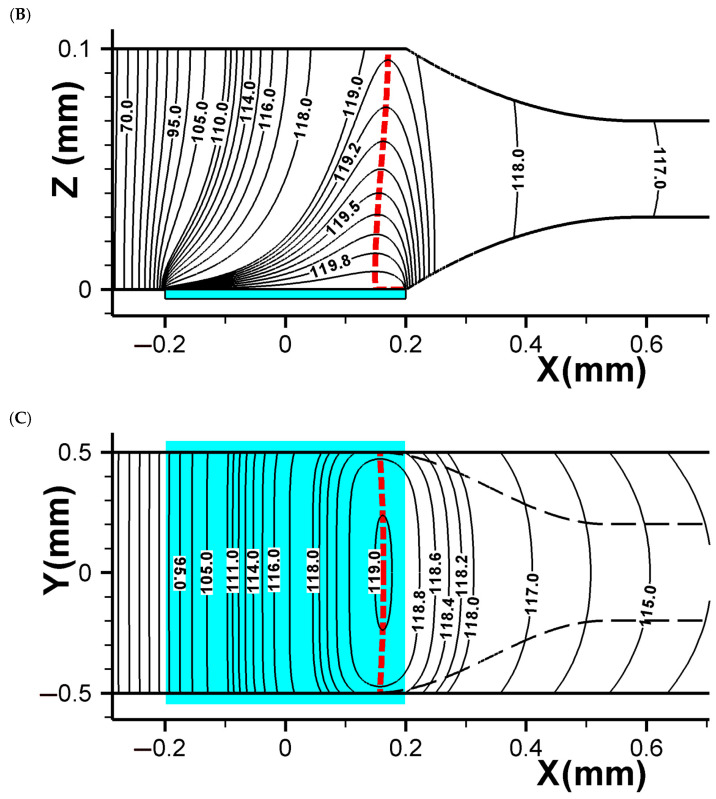
The stationary temperature distribution in the moving film in the middle longitudinal cross-section across the heater (**A**,**C**), and on the non-contact surface (**B**). (**A**,**C**) present uniform-size film, and (**B**) shows the film after the formation of the neck during drawing. The size of the film is 0.1 × 1 mm, the heater temperature is 120 °C, and the speed of the film movement is 10 cm/min relative to the heater surface.

Numerical simulation begins from the moment when moving a cold film comes into contact with the heater and film heating begins until the moment when the temperature distribution in the film becomes stationary. Examples of stationary temperature distribution are shown in Figure 5A–C.

The X-axis is directed along the film, and the Z-axis is perpendicular to the surface of heater. The heater is shown as a green rectangle in Figure 5. In the film of uniform cross-section (Figure 5A), the heat propagates from the heater to the opposite side of the film and forms a heated layer, which is shown in Figure 5 by the red vertical dashed line. Temperature homogeneity across the film in the Y direction is demonstrated in Figure 5B as a temperature distribution on the opposite side of the heater film surface.

The heated layer creates the conditions for drawing beginning and neck formation. The horizontal dashed lines in Figure 5A indicate the expected neck shape based on the position of the heated layer. Stationary temperature distribution after the neck formation is shown in Figure 5C.

**Figure 6:**
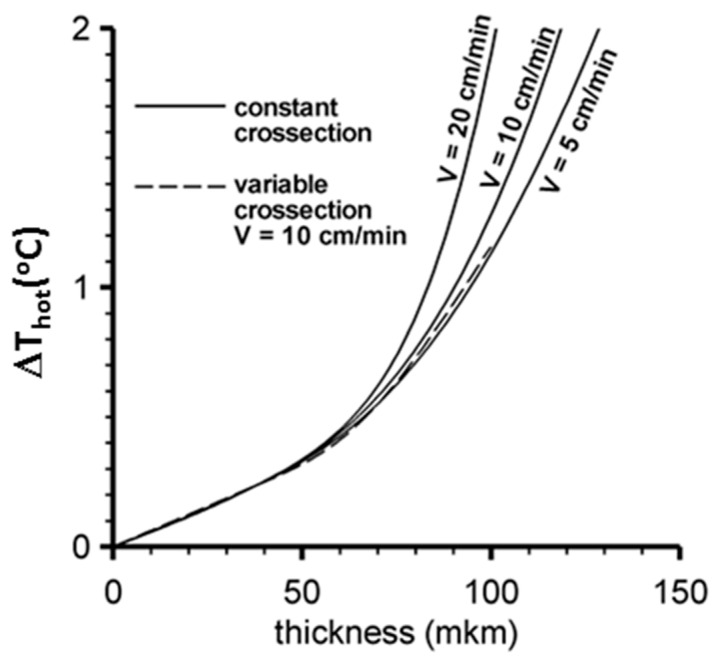
The dependence of ΔT_hl_ in the hot layer on the oriented film thickness, and on the speed of its movement along the local heater.

The calculated maximum temperature difference ΔT_hl_ in the hot layer between the contact and non-contact with the heater sides of the oriented film is shown in Figure 6 with a dependence on the film thickness and speed of the film movement along the heater.

After the cold film comes in contact with the heater, the film begins to warm up, and the temperatures of the contact and non-contact surfaces start to equalize. Finally, the temperature difference between them decreases to the value shown in Figure 6. Calculations show that the duration of the film’s heating is practically independent of its movement speed within the range of 5–20 cm/min and is approximately 250 ms for a film thickness of 50 µm, and about 350 ms for a thickness of 100 µm. The duration estimations in calculations are based on the time moment when the maximum temperature difference between the contact and non-contact surfaces of the film differs from the limit value T_hl_, shown in Figure 6, by less than 1%. At a speed of 20 cm/min, over 350 ms, the film will move a distance of about 1 mm, while the length of the films obtained in experiments is several centimeters. Thus, the temperature distribution in the oriented film during the experimental drawing can be considered to be stationary.

Let us consider the temperature difference between the contact and non-contact sides from the point of view of the concurrent strengthening and loss of strength. The orientational drawing might be considered as the Arrhenius-type process similar to the process of plastic deformation. Then, its rate v can be expressed by the equation which is similar to the lifetime τ expression:

(3) $$v=v_{0}\cdot e^{-\frac{U_{0}-\gamma_{v}\sigma }{kT}}$$

where *v*_o_ is a constant of the material equal to 10^12^–10^13^ s^−1^ [1,5], *U*_0_ is the chemical bond energy, γ*_v_* is the structurally sensitive factor, *σ* is the applied load, k is the Boltzmann constant, and T is the temperature of the oriented film. We assume that orientation drawing speed *v* is limited by the slowest of Arrhenius processes, i.e., by the bond breakdown process whose activation energy is highest.

Since the temperature on opposite sides of the film being drawn is different, it is necessary to determine the extent of the influence of this temperature difference (in our case, its magnitude is ~1 °C) on the on the plastic deformation of the film. Let us estimate the difference in drawing rate under the same load stress σ at different temperatures. The ratio of the drawing rates can be approximated from (3) by the following expression:

(4) $${\left.\frac{v_{1}}{v_{2}}\right|}_{\sigma =const}\approx e^{\left(\frac{U_{0}}{kT}\cdot \frac{∆T}{T_{1}}\right)}$$

Here, $T_{1}$ is the temperature on the non-contact side, and $∆T=T_{2}-T_{1}$ is the temperature difference between the two sides of the film. We neglected the small mechanical stress and assumed that ∆*T* is also small. Thus, the rate ratio is determined by the activation energy of the process. If we take the energy of C-C bond destruction as the activation energy E (*U*_0_) =6.4 × 10^−19^ J [40], then the rate ratio will be ≈ 0.73 at a temperature difference of only 1 °C.

The next conclusion from Equation (3) is that the lower the temperature of the material, the greater resistance it offers when drawn. Across the film section inside the hot layer, the stress from the drawing force will concentrate where the temperature is minimal. This conclusion is supported by the analysis of Equation (3). A consequence of the mass conservation law for the film passing through the neck is the equality of the drawing rates on the cold and hot sides: ${v_{1}}^{\prime }$*=* ${v_{2}}^{\prime }$. This produces the following equality:

(5) $$\frac{U_{0}-\gamma_{v}\sigma_{1}}{kT_{1}}\approx \frac{U_{0}-\gamma_{v}\sigma_{2}}{kT_{2}}$$

This means that when $T_{2}<T_{1}$, then $\sigma_{2}>\sigma_{1}$.

Thus, from one side, the lower temperature on the non-contact heater side of the film provides a lower rate of molecular rupture accumulation (see Equation (1)), and from the other side, the inhomogeneous temperature distribution over the film thickness entails inhomogeneous tensile stresses which are also included in Equation (1). This stress difference dominates over the effect of temperature difference. Therefore, one can expect the predominance of molecular destruction and the formation of macro- and microdefects in the “colder” surface layers on the film side that is not in contact with the local heater.

**Figure 7:**
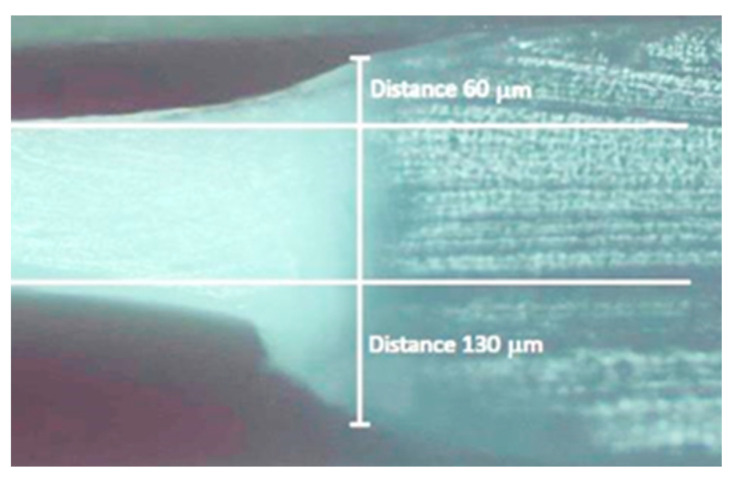
The optical micrograph of the side view of the longitudinal cut of the oriented film in the neck region. The contact heater side of the film is at the top. The non-contact heater side of the film is at the bottom.

Comparing Equations (1) and (3), it can be found that such a situation occurs if γ*_τ_* > γ*_v_*. Unfortunately, it is not possible to determine the values of γ*_τ_* and γ*_v_*. In this situation, experimental data can only confirm our suggestion. Indeed, at the side section of the oriented film (Figure 7), the difference in the film relief during neck formation can be clearly seen.

**Figure 8:**
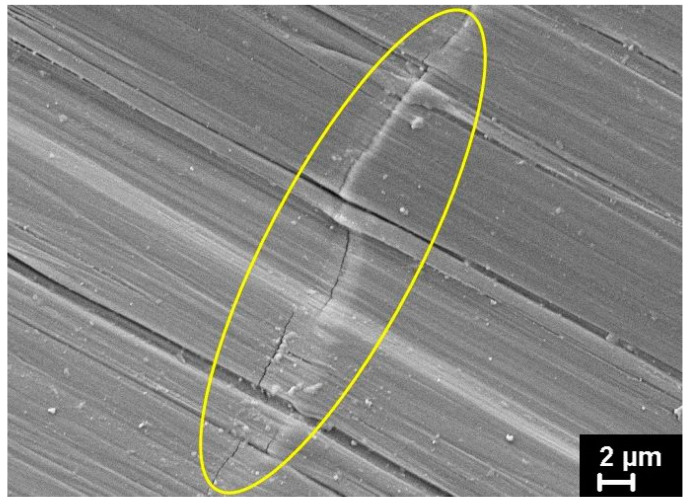
The microcrack on the boundary of a kink band on the non-contact heater side of oriented HDPE drawn with a draw ratio of 20.5 with a speed of 10 cm/min at 120 °C.

The transition to the neck zone occurs more smoothly on the contact side of the film than on the non-contact side, where a sharper decrease in thickness is observed. This indicates that the most synchronous unfolding of molecular folds is on the “cold” side of the film. Actually, the observed moment of transition of a non-oriented film into a macro-neck represents the merge of micro-necks gradually forming in the sample. However, this suggests that such a sharp transition to the fibrillar structure may be accompanied by multiple molecular scissions. They can play a negative role, namely the role of a trigger for the formation of large rotational defects, so-called kink bands, during further drawing. As is known, large entropic energy can accumulate in fibrils under the action of shear forces that arise during drawing due to the slippage of the fibrils over each other. It is released when individual defective fibrils are destroyed, which leads to the appearance of compressive forces in the direction of drawing and the formation of kink bands. The process of kink band formation, which ultimately leads to the fracture of the oriented film, is described in detail in a number of scientific papers [41,42]. It is important to emphasize that the appearance of kink bands begins on the “cold” side of the film, which is not in contact with the heater. As an example, one of these kink bands, at the border of which a microcrack has already formed, is shown in a yellow oval in Figure 8.

## 4. Conclusions

The main objective of this work was to evaluate the temperature distribution over the thickness of a polymer film subjected to oriented drawing using a local heater and to estimate the temperature difference between the heater-contacting and non-contacting sides of the film as a function of its thickness since the inhomogeneity of the temperature distribution over the film thickness can affect the mechanical properties of the resulting oriented polymer. Most attention is paid to the study of heat transfer at the first stage of orientation drawing, namely, at the stage of necking, when a film of initial thickness is fed to a local heater, and when the alpha relaxation temperature is reached in the crystal cores of nanolamellae, the initial folded lamellar structure is rearranged into a fibrillar structure under the action of an applied load (nake formation) as a result of the phase transition from a folded-chain crystal (FCC) to an extended-chain crystal (ECC). This process is accompanied by a decrease in the width and thickness of the oriented film with the formation of a neck at the transition point.

Numerical modeling of heat transfer in the film showed a non-uniform temperature distribution along the film thickness, depending on the speed of the oriented film feeding to the heater and on the thickness of the oriented film; the maximum temperature difference is reached between the contact (facing the heater) side of the film and the non-contact side, opposite to the contact side and being in the air. Since all kinetic processes occurring during oriented hardening depend exponentially on temperature and stress conditions (according to the Arrhenius equation), plastic deformation will proceed differently depending on the local temperature and local stress in the two near-surface layers of these sides of the oriented film.

The lower temperature on the non-contact side of the film compared to the contact side reduces the activity of thermofluctuation processes, which, according to Zhurkov’s theory, determine the rate of accumulation of molecular breaks. However, the inhomogeneous temperature distribution along the film thickness entails inhomogeneous values of tensile stresses; the stresses on the non-contact side are higher than on the contact side. According to the same Zhurkov theory, increasing stress decreases the activation energy and accelerates molecular rupture. It is assumed that the effect of inhomogeneity of stress distribution prevails over the effect of temperature inhomogeneity, which leads to the formation of macro- and microdefects in the colder near-surface layers.

It is suggested that defects formed in the colder near-surface layer will “work as a time mine” and lead to the formation of large rotational defects kink bands during further orientation drawing in the process of plastic deformation of the formed microfibrillar structure.

The formation of kink bands leads to premature rupture of the oriented material long before the limiting orientational elongations are reached and does not allow us to obtain oriented filaments with mechanical characteristics approaching the theoretical estimates. It is found that the most homogeneous temperature distribution over the film thickness, required, by default, for synchronous rearrangement of the non-oriented initial structure into a fibrillar one, is observed only for films less than 50 μm thick at a wide variation of the oriented film transport velocities through the heater. For thicker films, special attention should be paid to the selection of the drawing speed depending on the corresponding thickness of the oriented film in order to find the optimal temperature and force modes of orientation hardening used to obtain ultra-high-strength and high-modulus polyethylene yarns. Statistical confirmation of our hypothesis will require a large amount of experimental data on the detection of the moment of formation of kink bands as a function of temperature and drawing velocity.

## Data Availability

The original contributions presented in the study are included in the article, further inquiries can be directed to the corresponding author/s.
